# Reconsidering McKenzie’s Six Adventure Education Programming Elements Using an Ecological Dynamics Lens and Its Implications for Health and Wellbeing

**DOI:** 10.3390/sports8020020

**Published:** 2020-02-11

**Authors:** Jason King, Ashley Hardwell, Eric Brymer, Andrew Bedford

**Affiliations:** 1 School of Events, Tourism and Hospitality Management, Leeds Beckett University, Leeds LS6 3QN, UK; 2 Carnegie School of Sport, Leeds Beckett University, Leeds LS6 3QQ, UK; a.g.hardwell@leedsbeckett.ac.uk (A.H.); a.p.bedford@leedsbeckett.ac.uk (A.B.); 3 Discipline of Psychology, Australian College of Applied Psychology, Brisbane, QLD 4000, Australia; eric.brymer@acap.edu.au

**Keywords:** adventure education programming, Ecological Dynamics, adventure education, representative design

## Abstract

Two decades ago, McKenzie’s meta-analysis of literature provided six fundamental elements of adventure education programme design still used to guide research and practice today. While the value of McKenzie’s early work should not be underestimated, adventure education has undergone considerable changes. Adventurous activities are now available in urban and indoor contexts and used to facilitate a growing health and wellbeing agenda. The use of risk as part of adventure education programming has also been critiqued. This paper reflects on contemporary notions of adventure, risk and the emergent narratives emphasising the associated psychological benefits. The Ecological Dynamics framework, along with representative design delivery, are presented as a viable way of building on McKenzie’s work. Both consider how effective outcomes in adventure education programmes are achieved through designs that focus on the unique relationship between the individual and their environment. While McKenzie’s six elements recognise the importance of human relationships, Ecological Dynamics forefronts relational elements, not just between participants but, importantly, the task and the environment. Individual participant needs in relation to their everyday life therefore become the focus of adventure education expanding beyond the traditional long-standing narratives of risk and danger. Through these two important concepts, this paper advocates an approach to the design of adventure representative of a participant’s everyday environment. In this way, adventure education outcomes translate beyond the adventure-specific context and align more holistically with the needs of individual participants while also assuring emphasis on individual health and wellbeing.

## 1. Introduction

At the turn of this century, a meta-analysis of outdoor literature by McKenzie [1] succinctly captured six elements of adventure education programming (AEP) design that contribute to achieving programme outcomes. These elements were: the physical environment, activities, processing, the group, the instructor and the participants. McKenzie suggested that outcomes were dependent on how well the interaction between each of these categories aligned with programme goals. Physical environment referred to the use of unfamiliar outdoor settings to facilitate new perspectives on everyday environments. Activities referred to the quality of the activities designed into the programme and how well they provide mental, physical and psychological challenges and opportunities for mastery. Processing described the process of making sense of the programme by the participants and how learning in the adventure programme might be transferred to everyday life. The group element focused on the impact of group size, fellow participants and cooperation during learning. The instructor’s influence included how they interacted with the group and what they brought to the programme. The participant’s characteristics and capacity for autonomy were also considered important.

Underpinning these categories was the notion that effective adventure programme design suggested the inclusion of challenge and risk, for example, opportunities for emotional and social risk. Contemporary evidence suggests AEP is still based on activities designed to emphasise the impact of risk (physical and psychological), with little regard for nature and requirements of participants [2,3,4,5,6]. According to Wurdinger [7], it is risk, danger and uncertainty that differentiate AEP from other activities and impacts on the learning outcomes (see Brown and Beames [3] for a contemporary account of definitional issues). While the impact of McKenzie’s work should not be underestimated, as indicated by the fact that the six elements are extensively utilised in much of the current AEP practices [8,9,10], research into the outcomes and processes of AEP has increased and broadened exponentially since her original meta-analysis, which presents some challenges for understanding how best to apply the six elements and the value of the risk focus [3,11,12]. Furthermore, the last two decades has seen transformational developments in society through technological enhancement [13] and the indoorisation and urbanisation of adventure activities [4,14]. Adventurous education programming has embraced these advances, and contemporary programme design often includes technological adaptations and indoor activities [15,16,17]. Contemporary design is also rethinking the value of the human-nature relationship and the impact of AEP on health and wellbeing [3,18,19,20,21,22]. These transformations add to the notion that focusing on risk in AEP might be outdated, and the risk focus which underpins the six elements highlighted by McKenzie requires rethinking. It is possible a traditional interpretation of McKenzie’s six elements is limited in its capacity to support effective AEP design in contemporary contexts.

This paper revisits McKenzie’s six elements of AEP from a contemporary perspective and proposes that an interpretation and expansion of these elements through an Ecological Dynamics (ED) framework provides a grounded approach to effective AEP design and research. An ED interpretation of McKenzie’s six elements critiques the underpinning promotion of risk and danger as limited in its capacity to lead to AEP goals [23]. An ED approach also allows reframing of the dualistic presupposition that interactions between the six elements somehow act on the participant. Instead, ED promotes learning and behaviour as a relational notion that combines the characteristics of the learner, environment and task. ED recognises participants as active, embodied agents and emphasises the person-environment (social and physical) relationship as fundamental to behaviour and learning. Here, a sharp distinction must be drawn between what McKenzie sees as the importance of human relationships within AEP (participant, group and instructor, for example) and EDs emphasis on the relational, per se, which not only embraces the human but extends to relational elements of environment and task.

## 2. McKenzie’s Six Elements in Contemporary AEP

Careful consideration of McKenzie’s six AEP elements in the light of contemporary AEP contexts reveals a number of developments that stretch traditional interpretations. In this section, we highlight contemporary impacts in each element.

### 2.1. The Physical Environment

The proliferation of indoor and purpose-built environments is impacting on the variety of physical environments used in AEP [24]. For example, indoor environments for activities, such as climbing and skiing, allows for more efficient delivery of time-bound, task-oriented sessions. In the UK, for example, this has been compounded by the continued prevalence of packaged “off the shelf” outdoor and adventure delivery to schools through private organisations [25]. Outdoor environments continue to be sanitised, rationalised, commercialized and commodified [3,24,25,26,27] through the addition of purpose-built parks and centres, designed to provide opportunities for activities such as skateboarding, mountain biking, skydiving, surfing, rafting and kayaking. For instance, high ropes courses, zip wires and via ferrata all provide possibilities for convenient “unfamiliar physical environments” [1] (p. 20) where “seemingly” adventurous activities can take place. This has allowed a greater range of adventurous activity choices for AEP designers who are intent on emphasising risk management, danger limitation and the perception of unpredictable outcomes (though, in fact, outcomes are even more predictable). It also accentuates the importance of risky, dangerous, unfamiliar, even offbeat activities, which emphasise the centrality of the traditional risk narrative and allows particular providers greater “pulling power” over their potential “customers”. The importance placed on “cognitive dissonance” [8], also recognised by McKenzie [1], remains a central theme when designing and utilising contemporary physical environments in AEP contexts, perhaps with greater emphasis on perceived risk, rather than actual risk [28].

### 2.2. Activities

McKenzie’s [1] final remarks on this section are telling. She lists activity characteristics as being “holistic, incremental, and organized; that enable success, failure, goal setting, and choice; and that are chosen to produce specific outcomes” [1] (p. 22). However, she also suggests that “although these characteristics seem to be generally accepted as those that should be included in adventure education activities, there is remarkably little research to support this” [1] (p. 22). This alludes to taken for granted notions [29] of activity delivery with little empirical evidence to support the selection of activities for programmes, learning outcomes or participants. The choice of activities from which to select is now wide-ranging. It still seems that activities driving programme design and learning outcomes, or what a particular environment offers, is, at best, a secondary concern [30,31,32,33]. While place-based approaches are now more readily considered in programming [5] and can be strongly linked with the physical environment [30] and associated health and wellbeing benefits, the overarching default position for most programmes is still based on the notion that activities create cognitive dissonance for learners, which is the best way to meet programme outcomes [3,4]. Little substance has changed in the majority of practices where the focus still lies with novel activities creating a sense of danger and risk, allowing participants to feel physically and psychologically challenged.

### 2.3. Processing

McKenzie [1] (p. 22) defines processing as “the sorting and ordering of information” that contributes to programme outcomes. The three models of processing (or facilitation) alluded to are: minimalist intervention, where the “mountains speak for themselves”, guided facilitation by the leader and an approach with strong emphasis on metaphoric links [8]. The importance of processing is, once again, a taken for granted way of delivering adventure education outcomes. Often, it is the instructor who ensures each group member takes away what the instructor feels is important, because individuals “may not be able to make sufficiently clear connections between programme activities and their daily lives on their own” [34] (p. 25). There is strong consensus in contemporary writing on the importance of processing [34,35]. However, there is still little consensus about how learning in AEP transfers to everyday life contexts [36,37,38,39]. Critics also question the idea that processing after the event has the desired impact and instead call for a rethink of programme design [40]. Finally, processing is now regularly considered under reflective processes, and, while there is direct reference to reflection in AEP [41,42], a wider review of literature covering other educational contexts, where reflection is at the forefront of delivery, would be worthwhile (nursing and medicine, for example).

### 2.4. The Group

McKenzie [1] focuses on the importance of interpersonal “bonds” made by group members in AEPs. The “nowhere to hide” approach with a “warts and all” perspective on outcomes for group members [43] (p. 108) is supposed to facilitate deep and meaningful group connections over a short space of time. Group sizes, interactions and outcomes are deemed to be of considerable importance in AEP. However, McKenzie also recognised that knowledge about the processes involved is limited, and a better appreciation of the impact of groups is imperative. Given the wide participant remit of AEP (including women-only groups, disabled groups and hard-to-reach groups, as well as ages and diversities across the spectrum), it is surprising how little research has been conducted to address the issues raised, though there are exceptions [44,45,46].

Importantly, in more contemporary place-based practices [30], there has been a call for tailoring delivery to reflect group [5,39] or individual needs. However, the practice of activity-focused programmes that are underpinned by how best to exploit resources, such as time, staff and location, continue to dominate. The importance of income generation that drives many organisations now involved with AEP has also resulted in designs that reinforce efficiency (e.g., minimizing potential waste) rather than effectiveness (e.g., maximizing potential outcomes). While adventure tourism companies embrace the importance of bespoke experiences specifically tailored to the needs of participants [47] and regularly conduct market research to ascertain participant needs [48,49], such approaches have been slow to filter through to AEP.

### 2.5. Instructors

McKenzie [1] recognized the concentration of research efforts on understanding the link between “instructor styles, behaviours, and attitudes” (p. 24) and AEP outcomes. Interestingly, there was no mention of leadership. However, there is a plethora of writing on outdoor leadership elsewhere [8,50,51,52,53], which suggests the foundation skills alluded to in McKenzie’s paper still hold true today. McKenzie [1] (p. 24) glossed over the tangible instructor attributes (“technical, organizational, problem-solving, and decision-making skills”) and concentrated efforts on the hidden, more ethereal instructor issues (“biographical background, personality and interpersonal interactions”). She pointed to conflicting evidence in her study around gender and highlighted research emphasising the impact of instructor/participant relations. For the most part, research in this area has focused on determining leadership characteristics or behaviours linked to effective outcomes. Little attention has been paid to understanding the impact on learning outcomes (except perhaps in extreme situations) if sound practices are not applied [40,53].

### 2.6. The Participant

McKenzie [1] highlighted the role of participant gender on individual outcomes where stereotypical gender notions are reinforced. Males are reported to be interested in dominance, control, challenge and adventure, whereas females seek trust activities and spiritual development. A more recent study [54] suggested that AEP might have greater impact on the resilience of female students than male students. McKenzie’s [1] findings that sex stereotyping not only affects how men and women see each other but also how they see themselves might explain this finding. Allan et al. [54] suggested perceptions of resilience levels prior to an AE residential programme and ability to cope with the residential experience are overestimated in males and underestimated in females.

### 2.7. The Six AEP Elements in Context

Since McKenzie [1] first undertook her meta-analysis, some of the original six elements identified have been extensively researched, confirming the role they play in the creation of effective outcomes. Others, such as “processing”, still require further exploration. However, much of the original risk and danger narrative prevails.

Measuring the worth of programme outcomes for different participants remains difficult, particularly considering long-term benefits, because several variables are likely to impact on results [55]. Research has examined the impact of AEP on specific outcomes, such as resilience, through quantitative studies [56,57,58]. Research has also investigated the link between AEP and health and wellbeing across the globe, particularly among adolescents [59,60]. The impact of green space and the outdoor context on health and wellbeing has also been investigated [61,62]. Contemporary research has shown that the nature, scope and potential value of adventure education goes way beyond the traditional notion of expected AEP outcomes, allowing for more extensive personal benefits and general health and wellbeing to be accrued [25,33,62,63].

Importantly, scholars have also questioned the relevance of the risk-oriented approach to AEP that underpins much of McKenzie’s analysis [3,5,28,31,64] and highlights unfortunate cultural and historical assumptions implicit within the original meta-analysis. For example, while the participation of women in the outdoors continues to rise, outdoor leaders remain predominantly male, and programmes are often steeped in male machismo [65]. Forays into race and ethnicity prove even more problematic, as the outdoors remains essentially a white, male, predominantly middle-class space. While this paper does not extend to further exploration of these important issues, their impacts are significant. In response to many of these critiques, contemporary scholars have called for a bespoke learner-centred approach to AEP that emphasizes individual needs [3,54].

### 2.8. Building on the Foundations of McKenzie’s Work

The Ecological Dynamics (ED) framework provides a timely alternative to addressing the many points raised so far in a manner that suits twenty-first century living [66,67,68,69,70] by building on the foundations established through McKenzie’s six elements. From an ED perspective, the environment is much more than the conduit through which activities take place, and “decontextualized activity-based programmes” [71] (p. 9) are limited in their capacity to facilitate desired outcomes. Meaningful engagement by learners, considered active agents, with environments both natural and artificial can play a substantial role in supporting learning outcomes, enhancing participant health and wellbeing and providing intrinsic motivation for achieving personal goals [72]. The Ecological Dynamics perspective recognises a set of conditions that can be utilised in AEP to complement the work of McKenzie by addressing the contemporary issues raised, further extending our understanding of how effective AEP outcomes can be achieved.

## 3. Ecological Dynamics

Ecological Dynamics combines concepts from ecological psychology and the dynamical systems theory. Conceptually, ED recognises humans as dynamic, complex systems constantly interacting with other systems. It extends already familiar and well-developed physical, social, cognitive and emotional domain works [8] and understands these through the overarching, complex human-environment (social and physical) relationship. The human-environment relationship is key to understanding how behaviours emerge and suggests learner and environment are equally important in the relationship. Rather than the environment acting on a passive learner as if a separate external force, ED promotes a relational interaction. This is significantly different to the human relational element considered by McKenzie. Ecological Dynamics provides a more holistic relational element that goes beyond the identification of the human element within AEP. For example, the learner brings to the learning context height, limb length, emotional experience, culture, historical experience and so on. The environment is perceived in terms of function or possibilities for action (see below, under affordances), rather than in terms of its form. For example, surfaces are perceived as jumpable, or climbable, opportunities for shelter, communicating, food and so on. The relationship between person and environment guides behaviour and is time-bound. For example, an apple in a tree invites eating for an animal that can physiologically digest apples. In a human context, cultural background, hunger, preference, limb length, position of the apple, condition of the apple and so forth guide action and whether the apple is picked and eaten, left alone or even noticed.

Ecological Dynamics has been effectively employed to explain the learning process in a range of fields, including human movement science, psychology and physical education [73,74,75,76,77]. Research has demonstrated that ED effectively explains how lasting behavioural change emerges from the interactions between each individual, the environment and the task (or context). In this paper, we explore how the ED framework is ideally suited to guide effective AEP design, because it supports the idea that knowledge and behaviour emerge from the interactive relationship between an individual and the environment.

Ecological Dynamics is truly holistic, promoting an idea of learning as emerging from the relationship between an active, embodied, perceiving agent and the environment, which goes beyond the notion of passive holistic learning through the senses [77]. Instead, learning encompasses relational elements involved in the learning process. Each relational element is unique to each participant, therefore addressing the importance of a bespoke programme at the individual level. Designing learning opportunities using the ED framework invites participants to begin to address relationships with themselves, others, the task in hand and the physical environment. It also provides support for critiques on the value of designing programmes based on a one-size-fits-all approach and instead promotes a framework for learner-centred design. Key concepts from the ED framework useful for interpreting the six elements of McKenzie’s work are representative design, constraints, affordances and perception-action coupling.

### 3.1. Representative Design

Representative design is considered the most effective way of maximizing the potential for contextualized individual learning relevant for the everyday performance environment of the learner. Recognition of the need to design programmes that reflect everyday environments is already apparent in outdoor management development [78,79], yet not readily utilised in AEP. From an ED perspective, for learning to be useful in an everyday context, representative design is key. As noted above, learning emerges from the person-environment relationship. This idea differs from the information-processing model of learning that emphasises the human mind and the computer metaphor. Ecological Dynamics is also different from the behavioural approach, which focuses on the impact of the environment on the learner. The ED approach is also different from other approaches that emphasise the individual learner (including those that recognise the impact of the environment but only as an external influence acting on the individual—rather like a pill), arguing that such approaches reinforce organismic asymmetry (too heavily weighted on explanations that are individually determined). Ecological Dynamics frames learning as a process whereby the learner becomes attuned to meaningful information in an environment and adapts their behaviour accordingly. The learner is promoted as an active agent, and learning is an embodied process. For learning to be useful in the learner’s everyday environment, the design of the learning experience (environment and task) must be representative of the everyday performance environment. This does not suggest the environment needs to be physically the same but that key aspects of the everyday environment need to be effectively designed into the learning context. One implication of this idea is that if the learning context is not representative of the performance environment, the learner could attune to unhelpful information in the learning environment and, therefore, the learning context could be detrimental to the performance context. In ED, this is understood as one possible “rate limiter”. Other potential rate limiters, if representative design is not effective, include the leader, other members of group and the task. Interestingly, representative design also suggests that reflection will not counteract poorly designed learning experiences. However, well-designed learning experiences and reflection are powerful [40,53].

### 3.2. Constraints

Constraints encompass three main areas that influence and interact in a learning context: the individual, environment and task [55]. The term “constraints” relates to the characteristics of the individual, task and environment that interact to promote (or hinder) effective learning. From an ED perspective, as noted above, the individual is not seen as a passive recipient of stimuli via sensory mechanisms but rather an active organism continually attuning to information in the environment and adjusting behaviour to adapt to the environment or, on occasion, adapting the environment to achieve learning goals. As noted above, an individual brings unique dispositions to any context in terms of physical and psychological characteristics: culture, history, past experiences and so on, which lead to unique ways of interacting with an environment and the emergence of functional solutions to the tasks or challenges presented. However, the learning context can be manipulated to support effective learning by skilled leaders. For example, in AEP, the use of kayaking as part of a programme designed to facilitate group development with participants of different heights, limb lengths and experiences might be well-placed to have a fleet of kayaks of different sizes, shapes and volumes and paddles of different lengths, weights and feathering. Introductory sessions might encourage group experimentation through the completion of a short journey or similar, where groups of three guide each other, rather than traditional instructor-focused models. The leader can then observe and adjust tasks to help each learner and learning group achieve the desired goals. Interestingly, this also supports learner-centred approaches. The leader might also carefully consider the environmental characteristics appropriate for the learning context and manipulate as needed. For example, perhaps the light breeze could add challenge and support greater group work for some of the small groups, or perhaps the breeze might be too challenging for others.

In ED, perception is an active process [66], and this is crucial in understanding its dynamic transactional nature with other systems [80]. Importantly, the environment is conceptualized as both social and physical, and “experiencing the environment is not mental and subjective but understood relationally” [66]. The size, experiences, history and so on of the individual, combined with the opportunities in the environment, guide learning. If the learning designer does not consider this carefully, then, at best, learning will be weak and perhaps even detrimental to the performance context. In the example above, the use of kayaks and particular social and physical environments need to be carefully thought through in relation to learning goals. For example, is kayaking the tool for enhancing nature connection, health and wellbeing, resilience or another area, and, if so, how do the learning goals, individual constraints, task constraints and environmental constraints impact on the learning design?

### 3.3. Affordances

Affordances are opportunities for action that combine individual and environmental characteristics [81]. From the Gibsonian perspective, the notion of affordances signifies a recognition of a mutual relationship between the environment and perceiver. Gibson proposed the term to reflect opportunities for both good and ill; an affordance for good for one person might equate to an affordance for ill for another or the same person at different times. For example, bumps in the snow may prove to be an affordance for a serious injury for a novice skier, but, to an experienced skier, they provide an opportunity for honing skills or jumping [81]. This indicates affordances are not merely characteristics of the environment but relational notions that combine individual characteristics, abilities and capabilities (e.g., genes, physical ability, cultural background, personal history, skills, learning experiences, motivation and emotions), known as “effectivities”, with environmental characteristics. This relationship shapes the perception and action of affordances. To achieve maximum potential from affordances, ED recognizes constraints also exist in the individual, task and environment, interacting to shape perceptions and behaviours, cognitions and actions. These constraints can be manipulated to guide potential opportunities for action [82,83].

### 3.4. Perception and Action Coupling

Perception in humans is the process of orienting to and making sense of information in the environment that potentially leads to action. Whereas the traditional notion is sensory-based and emphasises how the individual makes sense of the passive process of sensory input for motivation and action, the ecological perspective is information-based and emphasises an active exploratory process. Gibson [83] identifies this as “the act of picking up information, moreover, is a continuous act, an activity that is ceaseless and unbroken”. The individual agent interacts with the environment in a continual perception-action cycle. Invitations in the environment, combined with the action capabilities of an individual, facilitate action possibilities. On occasion, affordances are perceived but not acted upon; on others, not perceived, and, on others, perceived and acted upon. The key being that the individual-environment relationship suggests a rich landscape of affordances that rely on the perception-action process to realise. Ecological Dynamics also proposes a view of perception and action that is different from the traditional notion where perception is the precursor of action. In ED, the coupling of perception and action means that action might lead to perception or the two might be so closely coupled it is impossible to determine an order. Perception is also described as direct, where meaning is already in the environment and detected through the person-environment relationship. For example, walking on rough ground can shape walking style as the agent (learner) learns to adapt to the environment; each step involves perception and action. On occasion, the act of placing the foot, for example, on an extremely rough patch, might lead to changes in gait. Characteristics of the environment are rich and directly perceived, and internal augmentation through mental models is not required.

## 4. AEP Design Using the Ecological Dynamics Lens

Practical examples often allow sense to be made of conceptual frameworks. To illustrate how the ED model can be applied in an AEP context, we will use the example of the first day of a five-day outdoor residential for first-year university undergraduate students embarking on their degree programme in Physical Education where team development activities are delivered. The learning outcomes for the overall programme are four-fold. First, the programme aligns with and offers opportunities to cover material associated with two first-semester modules that specifically consider academic outcomes linked to foundation academic skills; in this case, referencing and reflective practice. Second, the residential is designed to facilitate learning in effective group dynamics in a higher education setting, with focus on group assessments. Third, the residential experience is also designed as an important “ice breaker” for all students in the course to facilitate more effective relationships in the university context. Fourth, the programme is designed to support learning how to adapt in difficult and challenging circumstances. In this example, we will call this resilience the capacity to cope with adversity, as outlined by Connor and Davidson’s [84] five strands of resilience (personal competence, high standards and tenacity; trust in one’s instincts, tolerance of negative affect and strengthening effects of stress; positive acceptance of change and secure relationships; control and spiritual influences). Research indicates that resilience is fundamental to successful undergraduate completion [85,86].

### 4.1. Capitalising on the Individual-Environment Relationship

The implications for AEP design from the notions discussed above stem from the impact of the person-environment relationship in learning. For the most part, AEP explicitly aims to capitalise on unfamiliar outdoor settings to facilitate new perspectives in everyday environments. However, as discussed above, from an ED perspective, representative design is key to effective learning. Designing environments where learners can perceive and act upon affordances apparent in the everyday performance environment is essential. Learning communication or group effectiveness in an “artificial” outdoor context might only work if the affordances available reflect those in the everyday performance context. As such, effective AEP design requires a good knowledge of the performance context of each learner (e.g., the university environment). The physical and social environments in AEP might, for example, be manipulated to accentuate certain everyday contexts. Activity design might provide opportunities representative of effective group work in a university context. The vital message from ED is that learning goals and performance environments need to be aligned. Using the example above, an unfamiliar outdoor environment could be manipulated to provide the adverse conditions required to facilitate learning for resilience if the affordances required for effectiveness in an undergraduate setting are also available. For example, rather than designing activities that support physical competition and individualism, it may be better to design activities that support emotional support and collective appreciation. Activities that invite emotional support from fellow students and show their beneficial outcomes are likely to have greater impact on learning how to support each other in the university context than those that invite performance. The leader emphasising achievement goals in a climbing session or abseiling session is inadvertently supporting performance-oriented learning, perhaps even to the detriment of possibilities for emotional support. Conversely, an activity designed specifically to invite encouragement in difficult situations is likely to encourage similar behaviours in the performance context. It will also be important to ensure the individual learning that emerges from the AEP activities facilitates the capacity to attune to information in the environment in a manner that is appropriate to the university context.

Another important notion stemming from ED is that learning does not follow a one-size-fits-all process, because learning stems from the interaction between the learner and their environment. While AEP design should be representative of performance contexts, the rich landscape of affordances available to the learner will inevitably mean that learners will perceive and act upon a range of affordances. On occasion, this may not be helpful for the performance context. This means a leader will need to be continually vigilant and aware in order to adapt and develop the learning context as appropriate.

### 4.2. Responding to Individual Needs

Ecological Dynamics conceptualizes an individual as a complex system influenced by surrounding environmental characteristics [74,87]. Within the person-environment interplay, the physical and social environments shape opportunities for individual responses. Using the example above, students find themselves immersed in the beauty of the outdoors but also immersed in a social environment with a group they may have only known for two weeks. The perception and action of affordances is therefore shaped by each individual’s unique relationship with the social and physical environment. Some students may be very familiar with the outdoors or comfortable amongst strangers, whereas, for others, unfamiliar social groups or outdoor environments may be completely new and potentially rich with opportunities for anxiety. This may influence which affordances are perceived and acted upon. Recognising the uniqueness of each individual creates additional opportunities for potential personal success within AEP, inviting additional opportunities and learning benefits for participants. Individual characteristics, as previously noted, might filter the perception and action of affordances, so awareness of affordances and educating to these might be useful. The unfamiliar physical and social environment in the AEP can be representative of the unfamiliar physical and social environment in university if key affordances are designed into the AEP context. Developing resilience in this context needs to be useful for the university context. Awareness and educating to affordances for resilience development through the team development activities might require the discovery of individual feelings and opportunities. Individuals will need to appreciate the importance of adaptability and coping with difficult circumstances and develop the skills to adapt to the challenging tasks and environment in a manner suitable for individual constraints. As such, the AEP design might emphasise opportunities to perform in difficult contexts and the psycho-emotional impact/skills this invites. Personal tutors might work with students and become involved, not just in the team development activities but in the whole residential. All participants (including staff) see each other in completely new ways. Individuals become three-dimensional beings exposing layers of their multifaceted characters often hidden to the others. The tutor learns with the group and is responsible to, rather than for, their students [88].

Evidence from a growing number of disciplines suggests the physical environment plays a much greater role in adventure education than traditionally accepted [33,89,90,91]. These wider programme benefits become more pronounced when considered through the ED lens. The team development activity sites are carefully chosen in beautiful settings with rich opportunities for action invited by surrounding lakes, rivers and mountains. Beneficial transactions occur in the natural environment [66]. More specifically, health and wellbeing benefits have been reported, such as stress relief [91] and increased concentration [92]. ED recognises the relationship between the environment (physical and social) and the individual learner and emphasises the importance of facilitating all these relationships.

### 4.3. Moving away from Instructor Focused Activity

An important implication of the ED model is that instructors and other members of the individual learner’s group become part of the learner’s environment. This provides scope for moving away from a dominant instructor-centred approach and instead focuses on the relational importance between the individual students and the instructor as part of the learning environment. The student is therefore also part of the instructor’s environment, which suggests careful attunement to useful information afforded by each student. This demands a steep learning curve to begin with, but, over the five days, the instructor (and in the case above, personal tutor) can, first, offer affordances for good that may be usefully taken up by the student and, second, educate towards affordances not yet perceived. By focusing on individual students and their relationship to the specific environment, bespoke programming is possible. Therefore, key to the ED perspective in an AEP context is the instructor (and personal tutor) as an influencing factor in creating or educating towards affordances for individual learners.

Placing the instructor role within the context of the environment places more emphasis on the skills required to be effective. They are continually observing each participant, adjusting tasks to provide effective affordances for the action through which learning occurs. The team development afternoon (and indeed, the whole residential programme) becomes a microcosm of their three-year degree.

### 4.4. Academic Focus

Academic learning can filter into the team development activities that might also allow teams to begin relating to theoretical principles. During their delivery, students already begin to formulate relationships between theory and practice, how theory can be used to greater understand experience and how to articulate such thoughts in many different ways (perhaps physically, for example, through actually representing the different strands of resilience within a coloured wrist bands, or in other ways, such as discussions, reflective presentations and informal contact with others (including staff)). Tasks and actions are “representative” of the students’ everyday environment, because ED has been considered from the outset. Each task and action is purposefully aligned with what is expected in an undergraduate context. This reframes the role of the instructor (and the personal tutor) as the person uniquely attuned to each individual learner through a focus on individual participants, ensuring a more inclusive, holistic approach that accesses feedback from the physical, social and emotional environment to identify affordance opportunities.

### 4.5. Wellbeing Outcomes

Representative design suggests risk in AEP is only relevant if it is a key factor of the everyday environment. One of the greatest potential fears a student could face is the social and emotional risk of being with new people and not developing new friendships as the course progresses. ED, through attention to relational elements of the programme, would place emphasis on the health and wellbeing benefits of open and honest relationships with peers and also the unique contribution of the power of outdoor immersion. This on its own should be enough justification for tasks with a specific immersive, rather than risk-based, focus to be considered as the start of a week-long outdoor residential programme [28,37].

### 4.6. Practical Considerations

The final consideration for advocating such an approach is one of practicality. Representative design principles do not require wholesale change of activities with resultant costly implications. The process advocates modifying activities to achieve desired outcomes by fore-fronting the individual student-environment relationship. Representative design allows consideration of the alignment of activities specifically to the student experience while also providing a rich landscape of affordances for broader learning. In the example above, ED emphasises the relational element and the centrality of both personal tutor and outdoor instructor in ensuring affordances are maximized for each individual student. One way of doing this was to ensure how the uniqueness of each team development activity could be considered for each student. This was captured through depicting individual resilience through coloured resilience wristbands. This enabled a visual representation of Connor and Davidson’s [84] five strands of resilience for reflecting on by each student. Key programme changes only occurred in the area of resources for the wrist bands and enough lead-in time for personal tutors to become familiar with the team development tasks and the facilitation of these through resilience theory.

### 4.7. Real Reflection and Transfer of Learning

From the ED perspective, processing is also a task and should be designed to emphasise and ensure key affordances relevant to the students’ everyday world. The idea of representative design, while not new, is only just beginning to be adopted in AEP. Representative design builds on the idea of tailoring a programme beyond tried and tested activities and considering programming in its entirety as a task itself, allowing constant component modification in line with the fluid environment-person relationship to present affordances for good. Processing, or making sense of the experience, is presented as a key ingredient of successful AEP [8,52]. While effective representative programme design enhances perception and action coupling, processing is often the lubricant that refines learning. Evidence highlights that, for processing to be effective, the activity needs to be representative of the outcome for links to take place [93]. Individual students should be able to draw out skills relevant for a future context. A key ED concept is recognizing this link as perception-action coupling. In the example provided, this means the student needs to attune to meaningful information in a performance environment. Through representative design, the chances of learning being relevant for the everyday performance environment is enhanced.

### 4.8. Critical Evaluation

Section 4 provides practical considerations of how Ecological Dynamics is able to build on McKenzie’s work and move towards an individually focused approach to AEP with possible health and wellbeing outcomes. It addresses issues raised in Section 2, yet still recognises McKenzie’s six elements as important in AEP but accentuates holistic relational interactions rather than more one-dimensional human relations. However, the dynamic nature of AEP has to be recognised, and its complexities require many variables to align before meaningful outcomes may be realised. With this in mind, we acknowledge that, for ED to achieve maximum potential and successful outcomes, a combination of other factors requires addressing. For example, financial and logistical issues may require consideration. Philosophically, organisations may have to consider cultural changes for their successful implementations. Importantly, instructors relinquishing the role of leader, hard-skill developer and risk assessor to become the conduit for facilitation of the experience may prove problematic. Such design and facilitation changes could be difficult for well-established outdoor organisations steeped in traditional practices. Challenging existing, normative narratives is always difficult. Ecological Dynamics offers a framework to allow this to happen but only if the physical, philosophical and facilitation issues above are recognised and addressed. Research spanning the broad AEP community is needed to apply lessons learned to future programmes. The development of tools for programmers and practitioners is needed to assist in changing long-standing practices in AEP. One possible way of achieving this is by considering each of the elements of ED through empirical research and analysis.

## 5. Conclusions

This paper represents the first tentative steps in considering what happens when students embark on an adventure education programme using an ED approach. McKenzie [1] identified six possible elements involved in AEP outcomes. The ED framework forefronts the individual and their relational interactions with the environment and task and, therefore, reconceptualises her work. This represents a significant shift away from risk and danger-based AEP and towards bespoke programming that is truly holistic, extending learning beyond the senses and into the relational elements of a learning experience. Such an approach emphasises the instructor as a key player in facilitating AEP outcomes as part of the learner’s environment, rather than a leader of risk-based activities where risk and danger requires negation. Under an ED approach, instructors appreciate representative design, ensuring tasks are meaningful to the everyday context, and they progress the activities to maximise individual outcomes. They understand the outdoors to be a powerful environment which can offer a myriad of opportunities for contextualised immersive activities. ED represents a new way of considering AEP design relevant to each individual learner through representative design.
